# Hemodynamic forces enhance decidualization via endothelial-derived prostaglandin E2 and prostacyclin in a microfluidic model of the human endometrium

**DOI:** 10.1093/humrep/dez003

**Published:** 2019-02-21

**Authors:** Juan S Gnecco, Tianbing Ding, Caroline Smith, Jacky Lu, Kaylon L Bruner-Tran, Kevin G Osteen

**Affiliations:** 1 Women’s Reproductive Health Research Center, Vanderbilt University Medical Center, Nashville, TN, USA; 2 Department of Pathology, Microbiology and Immunology, Vanderbilt University Medical Center, Nashville, TN, USA; 3 Lead Contact; 4 Veteran Affairs Tennessee Valley Healthcare System, Nashville TN, USA

**Keywords:** endometrium, prostaglandins, shear stress, microfluidic, decidualization, reproduction, inflammation

## Abstract

**STUDY QUESTION:**

Does the uterine vasculature play a localized role in promoting stromal cell decidualization in the human endometrium?

**SUMMARY ANSWER:**

Our study demonstrated that hemodynamic forces induced secretion of specific endothelial cell-derived prostanoids that enhanced endometrial perivascular decidualization via a paracrine mechanism.

**WHAT IS KNOWN ALREADY:**

Differentiation of stromal cell fibroblasts into the specialized decidua of the placenta is a progesterone-dependent process; however, histologically, it has long been noted that the first morphological signs of decidualization appear in the perivascular stroma. These observations suggest that the human endometrial vasculature plays an active role in promoting stromal differentiation.

**STUDY DESIGN, SIZE, DURATION:**

Primary human endometrial stromal cells were co-cultured for 14 days with primary uterine microvascular endothelial cells within a microfluidic Organ-on-Chip model of the endometrium.

**PARTICIPANTS/MATERIALS, SETTING, METHODS:**

Cultures were maintained with estradiol and a progestin, with or without continuous laminar perfusion to mimic hemodynamic forces derived from the blood flow. Some cultures additionally received exogenous agonist-mediated challenges. Decidualization in the microfluidic model was assessed morphologically and biochemically. ELISA was used to examine the culture effluent for expression of decidualization markers and prostaglandins. Immunofluorescence was used to monitor cyclooxygenase-2 expression in association with decidualization.

**MAIN RESULTS AND THE ROLE OF CHANCE:**

A significantly enhanced stromal decidualization response was observed in the co-cultures when the endothelial cells were stimulated with hemodynamic forces (e.g. laminar shear stress) derived from controlled microfluidic perfusion (<0.001). Furthermore, the enhanced progestin-driven stromal differentiation was mediated via cyclooxygenase-2 and the paracrine action of prostaglandin E2 and prostacyclin. Altogether, these translational findings indicate that the vascular endothelium plays a key physiologic role during the early events of perivascular decidualization in the human endometrium.

**LARGE SCALE DATA:**

N/A.

**LIMITATIONS, REASONS FOR CAUTION:**

This report is largely an *in vitro* study. Although we were able to experimentally mimic hemodynamic forces in our microfluidic model, we have not yet determined the contribution of additional cell types to the decidualization process or determined the precise physiological rates of shear stress that the microvasculature of the endometrium undergoes *in vivo*.

**WIDER IMPLICATIONS OF THE FINDINGS:**

Identification of specific endothelial-derived prostaglandins and their role during endometrial reproductive processes may have clinical utility as therapeutic targets for reproductive disorders such as infertility, endometriosis, adenomyosis, pre-eclampsia and poor pregnancy outcomes.

**STUDY FUNDING/COMPETING INTEREST(S):**

This work was supported by the Veterans Affairs (I01 BX002853), the Bill and Melinda Gates Foundation Grand Challenges Exploration (OPP1159411), the Environmental Toxicology Training Grant (NIH T32 ES007028) and the Environmental Protection Agency STAR Center Grant (83573601).

**CONFLICT OF INTEREST:**

The authors report no conflicts of interest.

**TRIAL REGISTRATION NUMBER:**

N/A.

## Introduction

The human endometrium is a unique adult tissue that, in the absence of pregnancy or disease, continuously undergoes cycles of differentiation, breakdown and regrowth in response to changes in the ovarian steroids estrogen (E2) and progesterone (P4) (Noyes *et al.*, 1975). Importantly, the endometrial vasculature is equally subject to cyclic variations in ovarian steroid production (Cameron *et al.*, 1998). Following menstruation, endometrial regrowth is accompanied by vascularization of small arterioles and branching capillaries which provide oxygen and nutrients during tissue repair, growth and differentiation. The spatial and temporal expression of local inflammatory mediators, including cytokines, chemokines and eicosanoids, are also tightly regulated via direct and indirect mechanisms (Gellersen *et al.*, 2007; Singh *et al.*, 2011) and contribute to the normal regulation of the menstrual cycle (Jabbour and Sales, 2004; Catalano *et al.*, 2011; Gellersen and Brosens, 2014). Although numerous animal studies have illustrated the importance of the uterine vasculature in the reproductive tract (Kennedy, 1979; Rogers, 1996; Rogers and Gargett, 2001; Kennedy *et al.*, 2007), the degree to which the vascular endothelium contributes to endometrial reproductive processes in humans remains unclear (Hickey and Fraser, 2001; Gellersen *et al.*, 2007).

As a critical component of endometrial preparation for pregnancy, specialized stromal fibroblasts undergo a unique differentiation process called decidualization in response to E2 and P4. Proper decidualization is essential for the successful establishment and maintenance of pregnancy (Gellersen and Brosens, 2014; Garrido-Gomez *et al.*, 2017). This stromal cell transformation process is characterized by a transition to an epithelial-like cuboidal cell shape and the capacity to secrete pro-gestational proteins including prolactin (PRL) and insulin-like growth factor binding protein-1 (IGFBP-1) (Richards *et al.*, 1995). Decidualization has long been known to be a P4-dependent process; however, numerous studies suggest that the rise in ovulatory P4 is not the only factor necessary of initiating this process (Singh *et al.*, 2011; Gellersen and Brosens, 2014; Zhu *et al.*, 2014). In contrast to rodents, the onset of decidualization in humans and some old-world primates occurs in the mid-to-late secretory phase of the menstrual cycle in the absence of an implanting embryo (Gellersen and Brosens, 2014). Moreover, in humans, this stromal cell differentiation originates in cells immediately adjacent to the terminal spiral arterioles ~8–9 days after ovulation. Although this histologic observation was first recognized in the early 1950s (Noyes *et al.*, 1975), the mechanisms that promote this localized response have not previously been extensively investigated (Rogers, 1996; Gellersen and Brosens, 2014).

**Figure 1:**
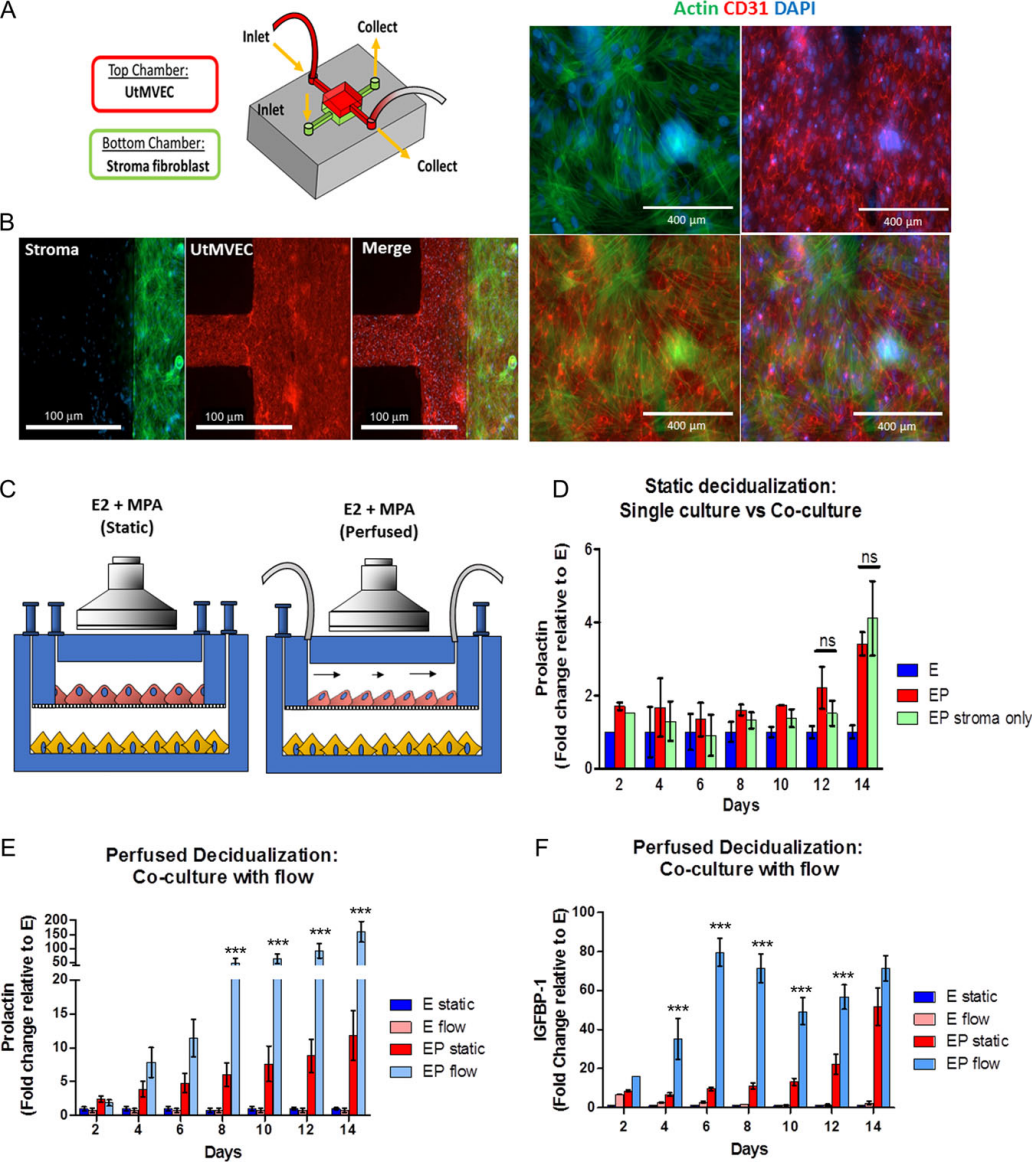
**Characterization of primary endometrial fibroblasts (stroma) and uterine microvascular endothelial cell (UtMVEC) co-cultures in a microfluidic model during decidualization under static and perfused conditions**. (**A**) Schematic of the perivascular stroma model using UtMVEC and primary endometrial stromal cells with perfusion. (**B**) Immunofluorescence staining reveals stromal (green, actin) and UtMVEC (red, CD31) monolayers maintained in co-culture exhibit appropriate cellular morphology during long-term cultures (scale bars= 100 μm (left panel) and 400 mm (right panel)). (**C**) Schematic of experimental design comparing static (i.e. gravity fed) vs flow perfusion during a 14-day experiment under the influence of a synthetic progestin medroxyprogesterone acetate (MPA). (**D**–**F**) Daily samples of spent media were analyzed for decidualization markers. (D) Stromal cell prolactin (PRL) secretion was similar in cells maintained as monocultures compared to static co-culture with perivascular endothelium. (E) PRL and (F) insulin like growth factor binding protein 1 (IGFBP-1) were significantly enhanced in the perfused model compared to static co-cultures. (*N* = 7) E=estrogen only (E2), EP = E2 + MPA, Flow = Perfused at 1 μL/min. *P* values, *<0.05; ** <0.01; ***<0.001.

In this report, we explored the proximal interactions between perivascular endometrial stromal cells and adjacent vascular endothelium in an effort to understand early events of decidualization in the human endometrium. We utilized a recently developed ‘Organ-on-a-Chip’ (OoC) microfluidic model of the human endometrial perivascular stroma to examine the crosstalk between stromal and endothelial cell types (Gnecco *et al.*, 2017). Importantly, this model of the perivascular stroma responds appropriately to hormonal changes and can be subjected to experimental laminar perfusion to mirror the hemodynamic forces derived from endometrial blood flow (Fig. 1A and C). The microfluidic design provides both spatial and temporal characterization of the cellular communication between endometrial cell types. Herein, our studies revealed that the perfused-vascular endothelium enhanced the sensitivity of stromal fibroblasts to P4, thereby enhancing the decidualization response. Moreover, the compartmentalized design of this microfluidic model demonstrated that hemodynamic forces regulate decidualization via a paracrine mechanism. Specifically, we identified that laminar shear stress modulates endometrial differentiation via endothelial-derived prostaglandins, specifically prostaglandin E2 (PGE_2_) and prostacyclin (PGI_2_).

## Materials and Methods

### Acquisition of human tissues and isolation of cells

The Vanderbilt University Institutional Review Board approved the use of human samples which were collected only after informed consent. Uterine tissues were collected from consented donors (ages 18–45) exhibiting predictable menstrual cycles and undergoing a hysterectomy for benign leiomyoma not associated with any other inflammatory ovarian or endometrial disease. Additional samples were obtained by pipelle biopsy from voluntary endometrial donors with no history of reproductive disorders nor on any hormonal contraceptive medication. Endometrial stromal cells were isolated by enzymatic digestion and filter separation as previously described (Osteen *et al.*, 1989) resulting in ≥ 95% purity as assessed by positive staining for vimentin. Stromal cells were passaged 1–5 times prior to *in vitro* assessment. Stromal cells were maintained in phenol red-free DMEM/F12 with 5% charcoal-stripped calf serum, 1 nM 17-β estradiol (E2, Sigma Aldrich, USA) and 1× antibiotic–antimycotic solution (ThermoFisher Scientific, USA) (stromal complete growth medium). Some stromal cell cultures were treated with 500 nM of the synthetic progesterone medroxyprogesterone acetate (MPA, Sigma Aldrich, USA) and/or 8-bromoadenosine-cAMP (0.5 mM, Sigma Aldrich, USA). Primary human uterine microvascular endothelial cells (UtMVEC) (Lonza, Cologne, Germany) were purchased and cultured as described by the manufacturer. Briefly, cells were cultured in endothelial cell specific media (EGM™-2MV BulletKit™ (Lonza, USA)) and maintained at 37°C in a saturated humidity atmosphere containing 95% air/5% CO_2_, and sub-cultured before reaching 60–70% confluence (approximately every 2 days) up to passages 8–10.

### Fabrication and assembly of microfluidic two-chamber device

The microfluidic organ-on-a-chip device was assembled as previously described (Gnecco *et al.*, 2017). Briefly, the complete device was fabricated using a polydimethylsiloxane silicone elastomer kit (PDMS, Sylgard® 184, Dow Corning, MI, USA) at a 1:10 ratio. Ports to access the microfluidic chambers were opened by punching 1/16′ holes in the top layer with a stainless-steel round punch (Integra Myltex, USA). Porous polycarbonate track etched (PCTE, 3 μm, 13 mm, Sterlitech, USA) were used as the semipermeable membrane to separate the two chambers. The PDMS layers and the membrane were oxygen-plasma treated (Plasma Cleaner 600 mTorr, 100 W, 45 s) and bonded together. For seeding and static maintenance, 500 μL Pyrex cloning cylinders (ThermoFisher Scientific, USA) were bonded with liquid PDMS to form reservoirs for the cell culture media.

### Cell culture within the in microfluidic device

UtMVEC were seeded and cultured using the procedure described elsewhere with a few experimental adaptations (Gnecco *et al.*, 2017). Briefly, the assembled, empty microfluidic devices were sterilized by UV exposure for 12 h. Poly-l-lysine (Sigma Aldrich, USA) was used to charge the PDMS for 20 min, washed with Dulbecco’s phosphate-buffered saline (DPBS, ThermoFisher Scientific, USA) and coated with a thin layer of collagen type IV (10 μg/cm^2^, Santa Cruz, USA) on both chambers then washed with PBS (3×). All reservoirs were aspirated using a 1 mL syringe (Monoject, Sherwood Medical, USA). Endothelial cells were seeded on the top chamber (2 × 10^6^ cells/mL) and stromal cells on the bottom chamber (1.5 × 10^6^ cells/mL) and allowed to adhere for a minimum of 30 min at 37°C. Endothelial cell growth basal medium (EBM-2) or complete stromal growth medium (350 μL) was added to the endothelial top chamber or stromal bottom chamber, respectively. Spent media was collected and replaced with fresh media every 48 h throughout all static experiments. Continuous laminar shear stress (perfusion) of the endothelial compartment of the microfluidic device was performed with a programmable PHD Ultra syringe pump (Harvard Apparatus, USA) with a 6 × 10 Multi-Rack adapter using 20 mL luer-lock syringes (Monoject, Sherwood Medical, USA) filled with EBM-2. Blunt-tip syringes (SAI, Infusion Technologies, USA) were connect to the inlet of the device via microbore Tygon® tubing (0.02'ID × 0.06'OD, Cole-Parmer, USA). Shear stress conditions were induced when the cells reached 60% confluence inside the chamber and continued for 14 days. The arbitrary rate of 1 μL/min was identified as the minimal experimental rate for stimulation of endothelial cells. Wall shear stress in the center of the chamber was calculated as described by Sung and Shuler (2012) by the chamber as a relatively flat channel (with width » height). The stromal cells were maintained under the same static culture conditions through all experiments. Collection of the effluent from perfused devices was performed using disposable polystyrene tubes (5 mL, ThermoFisher Scientific, USA) connected to the outlet of the devices via microbore tubing.

### Analysis of decidualization and prostaglandins

In static cultures, media was collected and replaced (300 μL) from all reservoirs every other day. Sample collection from perfused devices was performed by taking 600 μL from the collection tube connected to the vascular chamber outlet. The total volume dispersed by the pump was recorded for each experiment for downstream analysis. Collected effluents were frozen at −80°C until the termination of the experiment. Biochemical measurements of PRL and IGFBP-1, were performed using Human PRL and IGFBP-1 enzyme-linked immunosorbent assays kits (ELISA Duoset, R&D Systems), respectively. The ELISAs were performed according to the manufacturer’s instructions and expanded to 384 well format, using 23 μL per sample. Prostaglandins were measured using the PGE_2_ ELISA Kit – Monoclonal (Cayman Chemicals, USA) and urinary PGI_2_ ELISA kit (Enzo Life Sciences, USA) per the manufacturer’s protocol. Absorbance was measured using an absorbance microplate reader (GloMax® Multimode Readers, Promega, USA). Validation of diffusion of prostaglandins between the vascular (top) and stromal (bottom) chambers were confirmed. For perfused cultures, prostanoid production was calculated as a rate (pg/hr) rather than raw concentration to compensate for dilution associated with perfusion. In our system, media is not recirculated; thus, increased flow rates will result in increased total volumes collected over time. To overcome this issue, we converted from pg/mL to pg/h by calculating the concentration relative to the total volume of endothelial cell conditioned media (CM) collected, regardless of the length of time that it was perfused or the flow rate. Intra- and inter-assay coefficients of variation (CV) were as follows: PRL: intra-assay CV was 3.4% and inter-assay CV 5.4%; IGFBP-1: intra-assay CV was 4.1% and inter-assay CV 6.3%; PGE_2_: intra-assay CV was 4.2% and inter-assay CV 12.4% and PGI_2_: intra-assay CV was 2.9% and inter-assay CV 3.4%.

### Fluorescence microscopy and immunohistochemcial assessment

Formalin- fixed samples were subjected to fluorescence or immunohistochemical staining by standard methods as previously described (Gnecco *et al.*, 2017; Ding *et al.*, 2018) using the primary and secondary antibodies as described in Supplementary Table S1. Fluorescent slides were treated with ProLong® Gold antifade reagent with DAPI (ThermoFisher Scientific, USA) prior to adding the coverslip. For all studies, primary antibodies were omitted as a negative control. Slides were viewed using an Olympus BX51 microscope system and images captured using an Olympus DP71 digital camera.

### Conditioned media assays

Endothelial CM was collected using single chamber devices bonded to glass. UtMVEC were seeded at 1.5 × 10^6^ cells/mL and were cultured until they reached 80% confluence. BD-luer lock (12 mL, BD, USA) syringes were pre-loaded with media and warmed to 37°C prior to connecting to a Harvard Apparatus PHD pump and set at the desired flow rates; a collection tube was connected to the outlet of the single chamber device. A stepwise increase in flow rates was applied to the cells. After collection, the CM was centrifuged (300 RPM) to remove particulates and frozen at −80°C in aliquots. The CM was utilized by diluting 1:1 in fresh complete stromal media (complete DMEM/F12 media supplemented with E2 and MPA). For experimental CM, three single chamber devices with endothelial cells were serially connected and media pooled to adjust for dilution associated with perfusion. Static CM was acquired from cells grown and maintained in gravity-fed conditions. Endothelial media supplemented with hormones was used as cell-free controls. Inhibitory assays were performed with CM derived from endothelial cells perfused at 3 μL/min for 24 h supplemented with specific prostaglandin receptor antagonists: PGE_2_ receptor 2 (EP2) antagonist (AH6809, used at 20 μM Sigma Aldrich, USA), PGE_2_ receptor 4, (EP4) antagonist (AH23848, used at 20 μM Sigma Aldrich, USA), and PGI_2_ receptor (IP) antagonist (CAY10441, used at 10 μM Cayman Chemicals).

### Reagents used

A list of the reagents used in this study, with annotated concentrations, is provided in Supplementary Table S1.

### Statistical analysis

All experiments were performed from a total of seven primary control stromal cells derived from both endometrial biopsy donor and surgical hysterectomies, which exhibited differential sensitivity to steroid hormones. Consolidated data was individually analyzed as mean fold change relative to E2 (E) versus E2 and MPA (EP). Additionally, to accommodate the high variability between primary samples, the fold change relative to ‘E’ was calculated as the average of the ‘E’ between samples per day. Individual experiments were performed with at least two technical replicates. Data were analyzed using a Student’s *t*-test when comparing numerical variables between two groups and one-way analysis of variance followed by Bonferroni’s post-test for comparisons between more than two groups. Statistical analyses were performed using GraphPad Prism software; *P* < 0.05 was considered statistically significant.

## Results

### Continuous perfusion of the vascular endothelium enhances decidualization of stromal fibroblasts

The concurrent spatial and temporal changes that occur at the perivascular space suggest an interactive role for microvascular endothelial cells and endometrial stromal fibroblasts (Tabibzadeh, 1991; Horowitz *et al.*, 1993; Rogers, 1996). In order to examine the crosstalk of these two cell types, we adapted a previously established OoC model of the endometrial perivascular microenvironment (Gnecco *et al.*, 2017). We co-cultured primary stromal cells and UtMVEC in a dual-chamber microfluidic device separated by a semipermeable PCTE membrane. Endothelial cells were maintained within the OoC under static (e.g. gravity fed) or continuously perfused conditions (Fig. 1A–C), in order to assess the additional influence of hemodynamic forces on endothelial-stromal cell communication. OoCs were treated with EP over 14 days to induce decidualization (Fig. 1D–F). To identify the contribution of the vascular endothelium on this process in the OoC model, we monitored, morphologically and biochemically, the decidualization response of stromal cells that were cultured alone or with endothelial cells. In all experiments, we observed increased PRL secretion in cells treated with EP compared to E only; however, under static conditions, no statistically significant differences were apparent between stromal monocultures and the co-cultures (Fig. 1D). In contrast, decidualization was significantly enhanced in co-cultures when endothelial cells were maintained under continuous laminar perfusion at 1 μL/min throughout the duration of the 14-day experiment as observed by increased secretion of PRL and IGFBP-1 (Fig. 1E and F). Moreover, PRL expression was significantly enhanced in perfused co-cultures compared to perfusion of cultured stromal cells alone, further suggesting that these effects are mediated by the endothelium (Supplementary Fig. S1).

**Figure 2:**
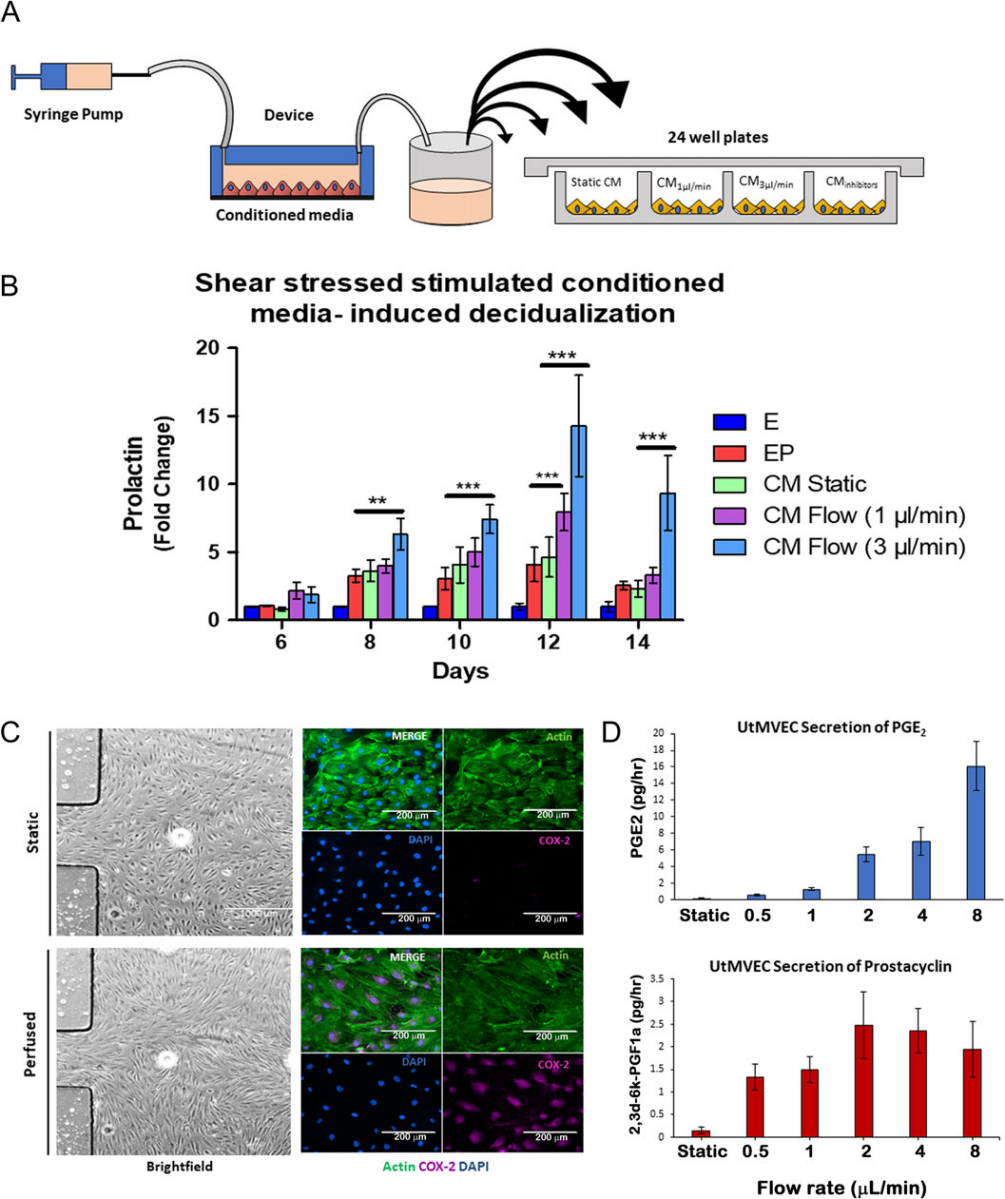
**Vascular endothelial cells enhance decidualization via a paracrine mechanism mediated by hemodynamic activation of COX-2 pathway**. (**A**) Schematic of experimental approach for perfused conditioned media (CM) assays. (**B**) Perfused endothelial CM accelerated PRL secretion on a flow-rate dependent manner compared to static endothelial CM (*N* = 7). (C and D) Characterization of shear stress activation of the UtMVEC in the microfluidic perfused model. (**C**) UtMVEC monolayer remodeling in response to flow conditions. Left hand panels show bright field images of cells cultured under static conditions (upper panel) and exposed to flow conditions (perfused at 1 μL/min; lower panel) within a single chamber microfluidic device (scale bars=1000 μm). Right hand panels show morphological remodeling and re-orientation of the actin cytoskeleton in direction of flow (actin, green) as noted by elongated endothelial cells and increased shear stress-induced expression of cyclooxygenase-2 (COX-2, purple) in the perfused (lower panel) compared to static (upper panel) conditions (scale bars=200 μm). (**D**) Validation of shear stressed secretion of endothelial-derived prostaglandins. Conditioned media from single chamber shear stressed endothelial cells was collected and measured for prostaglandin E2 (PGE_2_) and prostacyclin (PGI_2_) during stepwise increases in flow rates (*N* = 4). *P* values, *<0.05; ** <0.01; ***<0.001.

Next, we examined the effect of EP-primed CM obtained from monocultures of UtMVEC in single chamber devices that were perfused at increasing ranges of flow rates (Fig. 2A). Stromal cells that were supplemented with perfused endothelial CM, at 1 and 3 μL/min, exhibited significantly increased PRL secretion in a flow rate dependent manner compared to static endothelial CM (Fig. 2B).

### Endothelial–stromal co-cultures with laminar shear stress enhanced decidualization via COX-2 induction

Hemodynamic forces are well-known to stimulate endothelial cells and induce prostaglandin generating cyclooxygenase (COX) enzymes (Sandoo *et al.*, 2010; Triggle *et al.*, 2012; Russell-Puleri *et al.*, 2017). Specifically, COX-2 is the rate-limiting enzyme for the generation of terminal prostanoids. Therefore, we examined whether laminar shear stress forces induced the expression of the COX-2 signaling pathway in our microfluidic model (Vanhoutte, 1998; Lu and Kassab, 2011). Using single chamber devices, we stimulated UtMVEC monocultures under perfusion and observed the morphologic and molecular changes that are associated with shear stress stimulation. Specifically, at increasing flow rates, we observed endothelial re-orientation of the actin filaments toward the direction of the flow and a concomitant increased expression of COX-2 (Fig. 2C, Supplementary Fig. S2), Next, we demonstrated that the specific prostaglandins, PGE_2_ and PGI_2_ were secreted into the CM of perfused endothelial cells in a rate-dependent manner (Fig. 2D). Altogether, these findings suggest that laminar shear stress forces at increasing flow rates are experimentally inducing the prostaglandin pathway in our microfluidic model.

**Figure 3:**
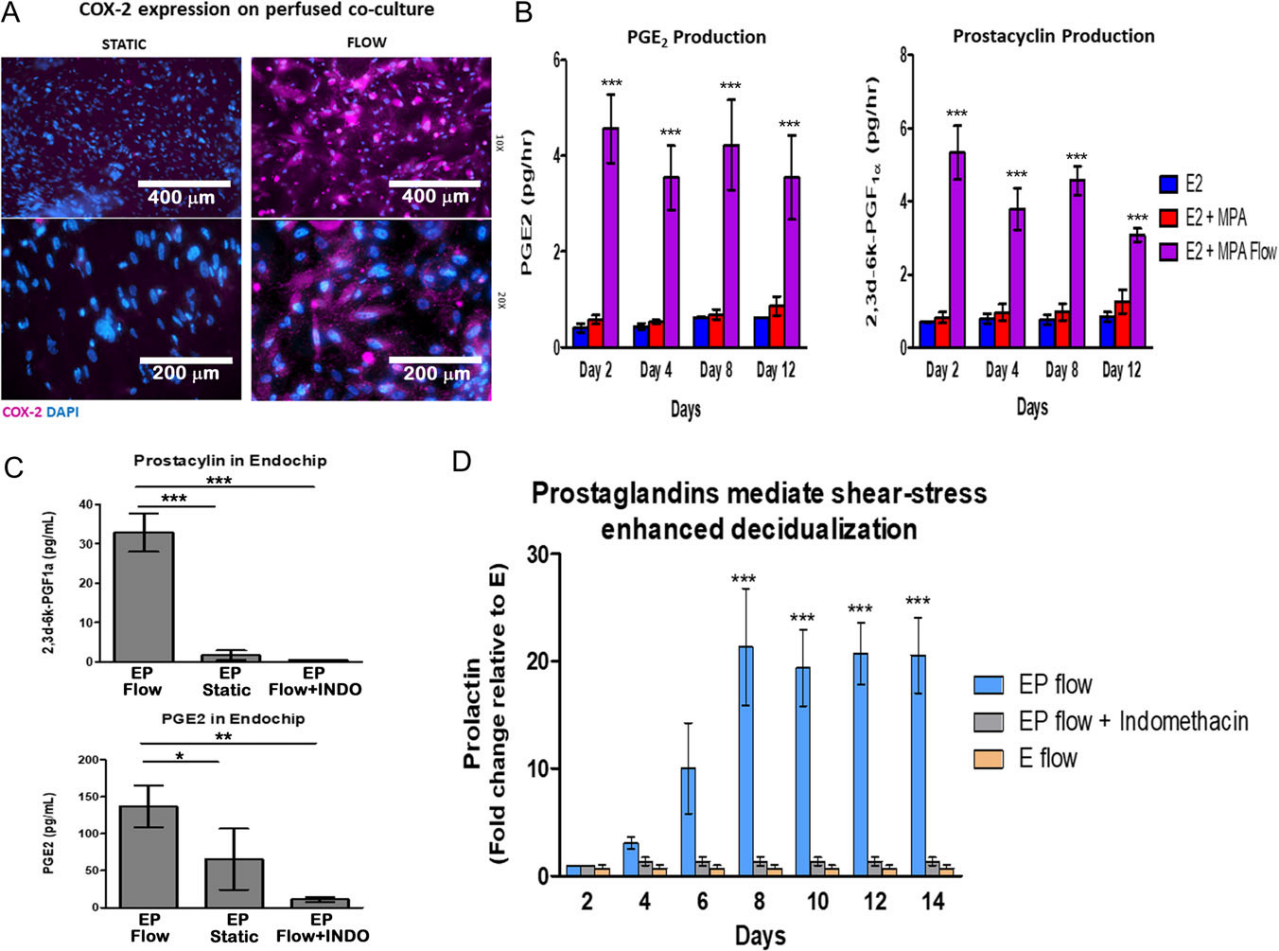
**The COX-2 pathway mediates perfusion-enhanced stromal decidualization in an OoC co-culture model**. (**A**) Immunofluorescent (IF) staining for COX-2 in perfused (1 μL/min) microfluidic co-culture devices demonstrates increased expression of COX-2 in the endothelial cells compared to static conditions (original magnification ×4 and ×20). (**B**) Subsequent secretion of prostaglandins such as PGI_2_ and PGE_2_ in microfluidic co-cultures under flow compared to static conditions. (C and D) Enhanced decidualization can be inhibited with administration of indomethacin (INDO, 100 μM) in the endothelial chamber of the co-culture model perfused throughout the 14-day experiment. (**C**) INDO treated co-cultures have reduced prostaglandins and (**D**) suppression of perfusion enhanced PRL secretion. *P* values, *<0.05; ** <0.01; ***<0.001.

Next, we conducted a 14-day experiment using the perivascular OoC co-culture of endothelial cells and stromal cells maintained under continuous perfusion and confirmed that the observed enhanced decidualization response was associated with expression of endothelial COX-2. As shown in Fig. 3A, immunofluorescence (IF) analysis of endothelial cell COX-2 protein expression was evident in the OoC co-culture. We also observed increased secretion of PGE_2_ and PGI_2_ in the effluent from perfused co-culture models throughout the 14-day treatment compared to static cultures (Fig. 3B). To confirm whether the prostaglandin pathway is mediating the enhanced decidualization in the perfused OoC co-culture, we examined the impact of indomethacin, a non-steroidal anti-inflammatory drug (NSAID). Treatment of endothelial cells with indomethacin suppressed the production of PGE_2_ and prostacyclin and resulted in a concomitant loss of the shear stress-mediated enhanced decidualization response (Fig. 3C and D). These findings confirm that COX-2 mediates the endothelial cell promotion of decidualization, supporting previous findings in animal models (Keys and Kennedy, 1990; Jabbour and Sales, 2004; St-Louis *et al.*, 2010).

### Endothelial-derived prostacyclin, and prostaglandin E2 promotes decidualization in the endometrial perivascular stroma

**Figure 4:**
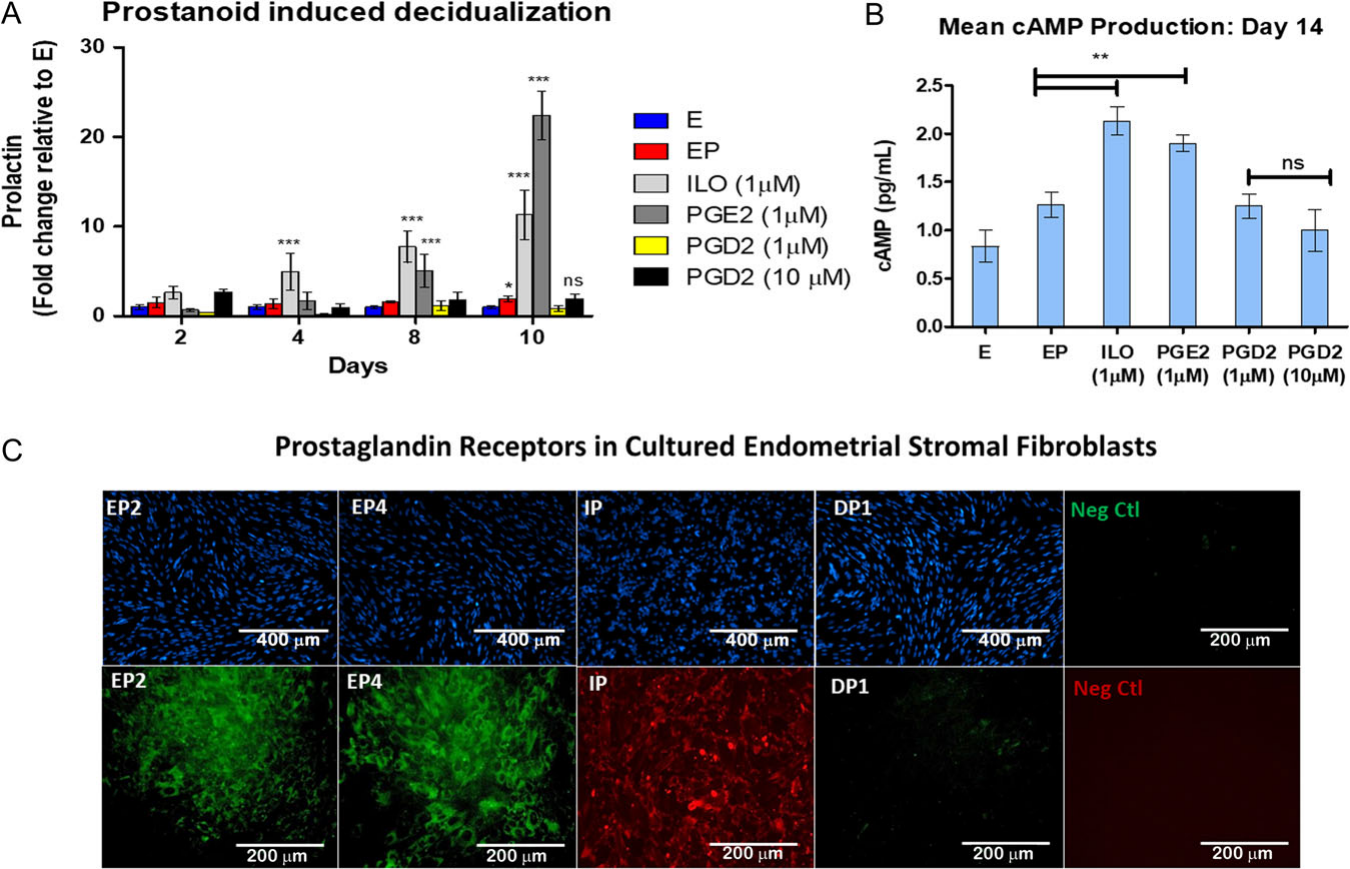
**PGI_2 _and PGE_2_ specifically enhance the decidualization response of stromal cells via activation of the cyclic adenosine 3′,5′-monophosphate (cAMP) pathway**. (**A**) Exogenous prostaglandins can enhance the decidualization process of endometrial stromal fibroblasts *in vitro*. The addition of PGE_2_ (1 μM) and a PGI_2_ analog Iloprost (ILO, 1 μM), but not prostaglandin D2 (PGD_2_, 1 or 10 μM), enhance the decidualization response. (*N* = 6 in duplicate). (**B**) cAMP production in stromal cells stimulated with exogenous prostanoids. (**C**) Representative immunofluorescence images of prostaglandin receptor expression in the cultured stromal cells. IP = PGI_2_ receptor; EP2, EP4 = PGE_2_ receptor isoforms 2 and 4, respectively; and DP1 = PGD_2_ receptor. (*N* = 6) in duplicate. *P* values, *<0.05; ** <0.01; ***<0.001.

Prostanoids are separated into four major types of bioactive prostaglandins: PGE_2_, PGI_2_, prostaglandin D2 (PGD_2_) and prostaglandin F2α (PGF_2α_). Not surprisingly, these prostanoids hold multifaceted roles in regulating both homeostatic functions and pathogenic processes. PGE_2_, PGD_2_ and prostacyclin are known to be potent activators of intercellular cAMP and have previously been implicated in reproductive function (Frank *et al.*, 1994; Brar *et al.*, 1997; Brosens *et al.*, 1999; Mizuno *et al.*, 1999; Smyth and FitzGerald, 2002; Gellersen and Brosens, 2003; Regan, 2003; Gellersen *et al.*, 2007). Thus, we examined the response of human endometrial stromal cells to these prostanoids to identify the specific endothelial-derived terminal prostaglandins that enhanced decidualization in our system. EP media supplemented with exogenous PGE_2_, PGD_2_ and iloprost (ILO), an analog for PGI_2_, was administered to cultured endometrial stromal cells *in vitro*. We observed that both PGE_2_ and ILO significantly enhanced PRL secretion (Fig. 4A). As expected, this was associated with increased stromal cell cAMP production (Fig. 4B). In contrast, PGD_2_ treatment did not promote decidualization nor production of cAMP in similarly cultured cells. Moreover, as expected from the literature (Banu *et al.*, 2008; Catalano *et al.*, 2011; Lee *et al.*, 2013), cultured stromal cells in our studies were confirmed to express the receptors for PGE_2_ receptors (EP2, EP4), and PGI_2_ receptor (IP), but not PGD_2_ receptor 1 (DP1) (Fig. 4D), likely explaining the lack of response for PGD_2_.

**Figure 5:**
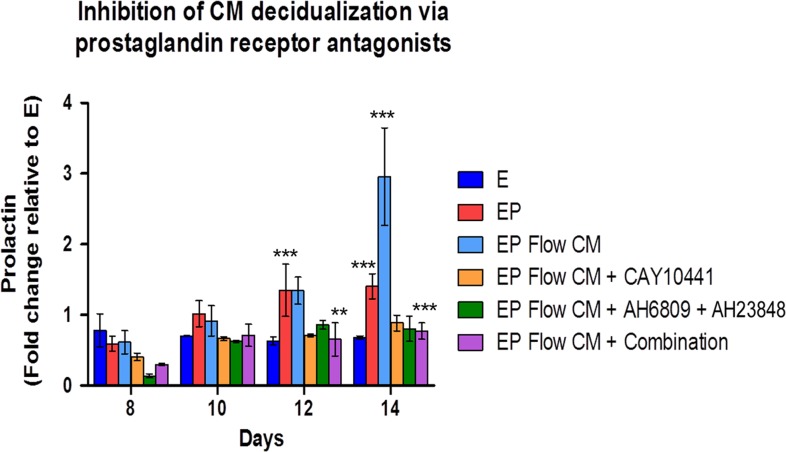
**Shear stress-induced endothelial-derived prostaglandins enhance decidualization via stromal prostanoid receptors**. Accelerated decidualization was observed in a flow rate dependent manner using conditioned media from perfused endothelial compared to treatment with conditioned media from cells cultured without perfusion. CM-induced decidualization from shear stressed endothelial cells (3 μL/min) can be blocked with co-treatment with prostanoid receptor antagonists. CAY = IP inhibitor (CAY10441, 10 μM), EP2 and EP4 = specific PGE_2_ receptor inhibitors, (AH6809, AH23848, respectively, 20 μM), Combination = EP2 (20 μM) + EP4 (20 μM) + CAY (10 μM). *N* = 6, in duplicate. *P* values, *<0.05; ** <0.01; ***<0.001.

Furthermore, CM-induced stromal cell differentiation could be blocked with the addition of specific prostaglandin receptor antagonists. We observed strong suppression of decidualization by blocking IP or EP2 and EP4, and a modestly additive effect when all inhibitors were used in combination (Fig. 5). Taken together, these findings suggest a robust temporal role of prostacyclin and PGE_2_ during the initiation of decidualization in the human endometrium.

### Temporal co-localization of COX-2 and endometrial decidualization in whole human tissue

**Figure 6:**
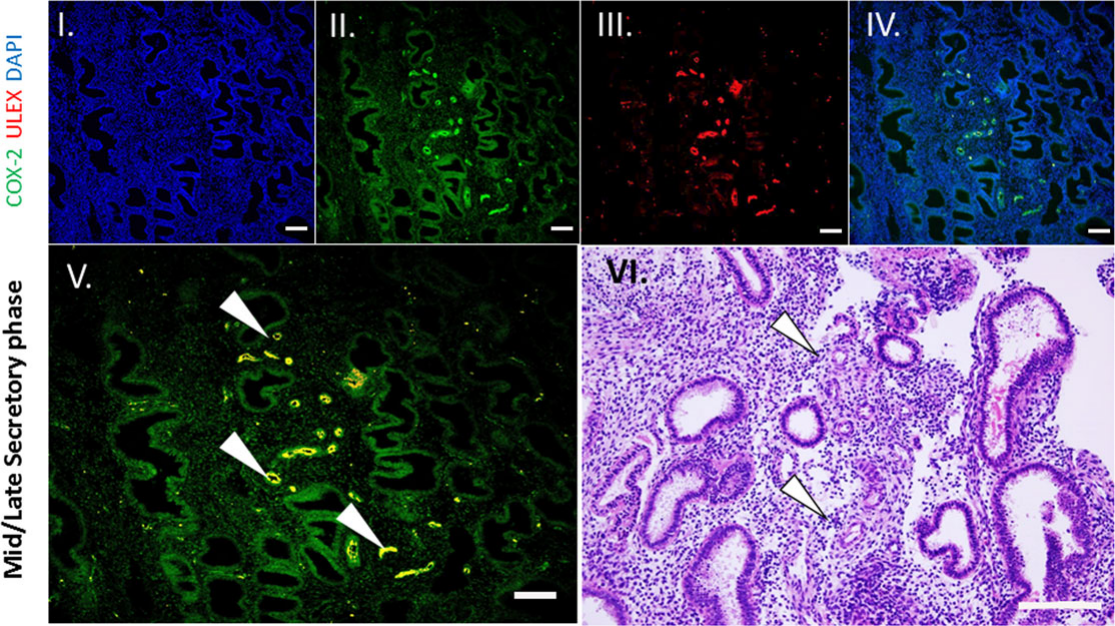
**COX-2 co-localizes to the human spiral arterioles in the secretory phase of the menstrual cycle**. Histologic section of human endometrium beginning to decidualize at perivascular sites. (I) 4′,6-diamidino-2-phenylindole (DAPI, II) COX-2, (III) Ulex europaeus agglutinin I (ULEX) was used to identify endothelial cells, (IV) Merge of COX-2 and DAPI. (V) Merge of all images. Arrowheads depict co-localization of staining (yellow, ×20). (VI) Haematoxylin and eosin stain (H&E) of mid secretory phase human endometrium exhibiting decidualization of the perivascular stroma (arrows). All scale bars = 50 um.

Lastly, we demonstrated a similar role of COX-2 activation in *ex vivo* human endometrium (Noyes *et al.*, 1975). Endometrial surgical samples that clearly exhibit decidualization at perivascular sites were examined histologically via IF for COX-2 and the endothelial cell marker Ulex europaeus agglutinin I (ULEX). These results revealed co-localization in the vascular endothelial cells in the human endometrium from the secretory phase, but not extensively in the proliferative phase (Fig. 6; Supplementary Fig. S3). Importantly, perivascular decidualization of these same tissues was observed only in cells surrounding the spiral arterioles, while stromal cells distal to vessels did not exhibit morphologic changes (Fig. 6, VI). In comparison, residing immune cells and epithelial glands predominately expressed COX-2 in proliferative phase tissues, but do not promote stromal cell decidualization, suggesting a cycle dependent expression of specific terminal prostaglandin synthases and their respective receptors (Milne *et al.*, 2001; Battersby *et al.*, 2004; Catalano *et al.*, 2011).

## Discussion

A large body of *in vivo* and *in vitro* studies has defined the role of hemodynamic forces in the regulation of vascular function (Li *et al.*, 2005; Davies, 2009). Specifically, laminar shear stress can stimulate endothelial cells via mechanoreceptors that activate intracellular signaling pathways that modulate numerous intercellular responses (Vanhoutte, 1998; Lu and Kassab, 2011; Russell-Puleri *et al.*, 2017). At this juncture, animal studies have provided the best evidence that vascular events are essential for reproductive function (Kennedy, 1979; Li *et al.*, 2005; Kennedy *et al.*, 2007; Lockwood *et al.*, 2009; Bany and Hamilton, 2011; Singh *et al.*, 2011), yet only a few groups have attempted to model the human endometrial vasculature (Albrecht *et al.*, 2003; Goodwin, 2007; Fan *et al.*, 2008; Martins-Green *et al.*, 2008; Goddard *et al.*, 2014). As such, there is currently a limited understanding of the functional role of hemodynamic forces within the endometrial vascular bed on uterine function. Unfortunately, traditional 2D *in vitro* cultures are not capable of mimicking the hemodynamic events occurring in the endometrial microenvironment (Papp *et al.*, 2000; Albrecht *et al.*, 2003; Park *et al.*, 2003; Bissell and Labarge, 2005; Wikswo *et al.*, 2013; Bhatia and Ingber, 2014). To overcome these limitations, we utilized an OoC microfluidic model of human endometrial perivascular stroma as an experimental tool to test hemodynamic perfusion on endometrial decidualization. Our results demonstrate not only the utility of our experimental model to examine the role of hemodynamic forces in the human endometrium, but also determined that the microvascular endothelium contributes to reproductive success. Nevertheless, additional studies will be required to fully mimic the physiological hemodynamic rates present in the human endometrium.

**Figure 7:**
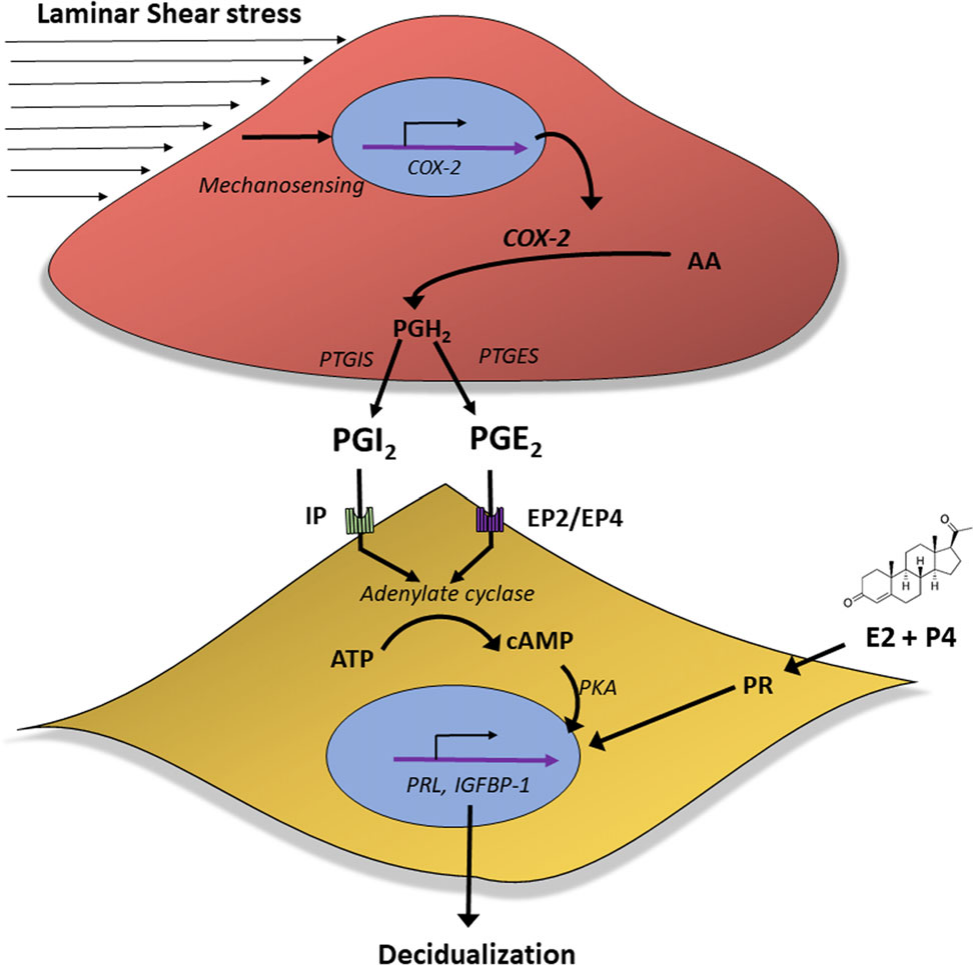
**Proposed mechanisms driving localized decidualization in the cycling human endometrium**. Prostaglandins can enhance the decidualization process in the human endometrium. *In vivo*, decidualization originates in the stroma surrounding the vascular endothelium. Activation of endothelial cells by physiological cues such as shear stress forces can induce the prostaglandin pathway in endothelial cells and promote the secretion of prostaglandins. Specifically, PGI_2_ and to a certain extent PGE_2_, but not PGD_2_, can accelerate decidualization in adjacent endometrial stromal cells via their respective prostaglandin receptors. These events promote the enhanced response to progesterone (P4) via cAMP activation that lead to increased decidualization.

The successful establishment of human pregnancy is dependent on the ability of P4 to initiate the decidualization response within the stromal compartment of the endometrium (Singh *et al.*, 2011; Gellersen and Brosens, 2014). The relatively long delay between exposure to post-ovulatory P4 and the onset of spontaneous stromal decidualization suggests that locally produced factors, in addition to P4, are necessary for decidualization (Gellersen and Brosens, 2014). However, aside from cAMP, the specific identities and roles of these factors remain speculative. Herein, our findings demonstrate for the first time that continuously perfused vascular endothelial cells, even at relatively low flow rates, enhance the decidualization of human endometrial stromal fibroblasts via a paracrine mechanism. We demonstrated that specific shear stress-induced prostaglandins are centrally responsible for mediating the focal process of perivascular decidualization (Fig. 6). Additionally, blocking COX-2 expression with NSAIDs or blocking specific prostaglandin receptors downstream suppressed this effect (Figs 3 and 5). Based on our data, we propose that in the human endometrium, hemodynamic forces act on the endometrial vasculature to promote expression of specific prostaglandins that induce endometrial stromal cell production of cAMP, thereby enhancing P4-associated stromal cell decidualization (Fig. 7). Our proposed mechanism of action is supported by previous *in vivo* animal models in which pharmacological or genetic suppression of the COX-2 pathway can delay or block localized decidualization and implantation in a variety of species (Keys and Kennedy, 1990; Hamilton and Kennedy, 1994; Kennedy *et al.*, 2007; Clark and Myatt, 2008). In turn, exogenous addition of specific prostaglandins in similar models acts to directly reverse the effects of blocking COX-2 (Holmes and Gordashko, 1980; Garg and Chaudhury, 1983). Taken together with our human data, these findings in relevant species may help explain the association between chronic NSAID use and reduced fertility in reproductive age women (Akil *et al.*, 1996; Livshits and Seidman, 2010).

The prostanoid family includes numerous short-lived, lipid soluble inflammatory mediators which exert diverse biological actions and hold multifaceted roles in both homeostatic functions as well as mediating pathogenic inflammatory responses (Brar *et al.*, 1997; Gellersen and Brosens, 2003; Sandoo *et al.*, 2010; Singh *et al.*, 2011; Triggle *et al.*, 2012). Prostaglandins exert their effects via G protein-coupled receptors (GPCRs) (Ricciotti and FitzGerald, 2011) and regulate a range of intracellular signaling pathways within the reproductive tract. Most notably, activation of EP2, EP4, IP and DP1 receptors can induce adenylyl cyclase activity and increase intercellular concentrations of cAMP, a known modulator that is required to promote decidualization (Jonsson *et al.*, 1979; Holmes and Gordashko, 1980; Lim *et al.*, 1997). In this report, we demonstrated that PGE_2_ and PGI_2_, but not PGD_2_, significantly enhanced decidualization via increased intercellular cAMP. We found that prostacyclin consistently promoted a more rapid decidualization response compared to PGE_2_, albeit at a lower magnitude. Although prostacyclin may exert its effects through alternative nuclear receptors such as the peroxisome proliferator-activated receptor (PPARγ) (Lim *et al.*, 1997; Dussault and Forman, 2000), we demonstrated that its effects can be blocked by inhibiting the stromal cell IP receptor. Substantial evidence, primarily using animal models, supports our findings that prostaglandins related to uterine function are critical for reproductive success in humans, specifically prostacyclin and PGE_2_ (Vesin *et al.*, 1979; Frank *et al.*, 1994; Hamilton and Kennedy, 1994; Kennedy *et al.*, 2007). Furthermore, our findings revealed the identity of key prostaglandins activated during early decidualization, potentially offering an opportunity to target these molecules for therapeutic development. Clinically, targeting specific terminal prostaglandin species, may provide a putative non-steroidal solution to alleviate pain and chronic inflammation for certain reproductive disorders, while maintaining or restoring endometrial receptivity (Lee *et al.*, 2013; Laux-Biehlmann *et al.*, 2015; Rudzitis-Auth *et al.*, 2018). Thus, revisiting the role of specific prostaglandins and their receptors that may be critical for reproductive function of both the gravid and non-gravid uterus is appropriate.

Like all model systems, our model and studies presented in this report have several limitations. Unfortunately, the exact shear stress rates within the microvasculature of the human endometrium are difficult to precisely calculate and have been closely linked to steroidal changes during the menstrual cycle (Raine-Fenning *et al.*, 2004; 2004; Osol and Moore, 2014; ; ). Our model was designed as experimental tool to test ranges of relative perfusion flow rates, yet further work on specific flow rates is required. Nevertheless, impaired endometrial blood flow has been associated with unexplained infertility (Raine-Fenning *et al.*, 2004). Interestingly, dysregulated endometrial vascular function and associated alterations in paracrine factor production have also been observed in women with reproductive disorders such as endometriosis and pre-eclampsia (Smith *et al.*, 1981; Cameron *et al.*, 1998; Jabbour and Sales, 2004; Achache *et al.*, 2010; Garrido-Gomez *et al.*, 2017). The timing, stability, relative concentrations of prostaglandins and the expression of their receptors, within the endometrium is likely critical to determine stromal responses (Clark and Myatt, 2008). Importantly, while a number of cells, including endometrial epithelial cells, produce prostaglandins, prostacyclin is a known to be a primary product of vascular endothelial cells (Schror, 1985), suggesting a potential role in regulating reproductive function in the perivascular space (Smith and Kelly, 1988; Lim *et al.*, 1997; Hickey and Fraser, 2001; Russell-Puleri *et al.*, 2017). Nevertheless, the contribution of additional endometrial components such as epithelial cells and macrophages must be considered, and it will be necessary to investigate in detail the redundancies of prostaglandin signaling in the endometrium. Lastly, herein, we demonstrated that the hemodynamic induction of prostaglandins by endothelial cells enhanced decidualization of stromal cells (Fig. 3). However, it is interesting to note that we have previously found that induction of prostaglandins by pro-inflammatory cytokines can act to suppress decidualization (Gnecco, JS and Osteen, KG, unpublished observation), suggesting that the context of COX-2 expression linked to a pathogenic stimulus is different from physiological stimulation via hemodynamic forces. The divergence of physiologic and pathogenic pathways will need to be explored further in future studies.

## Supplementary Material

Supplementary Figure 1

Supplementary Figure 2

Supplementary Figure 3

Supplementary Table 1
