# Supplementary material for: Hemodynamic forces enhance decidualization via endothelial-derived prostaglandin E2 and prostacyclin in a microfluidic model of the human endometrium
Source: Hum Reprod. 2019 Feb 21;34(4):702–14. doi: 10.1093/humrep/dez003 (PMC6443116; doi:10.1093/humrep/dez003)
Supplement: Supplementary Figure 1 [file dez003_supplementary_figure_1.pdf]

Human Reproduction, 2019
doi:10.1093/humrep/dez003

human reproduction

SUPPLEMENTARY DATA

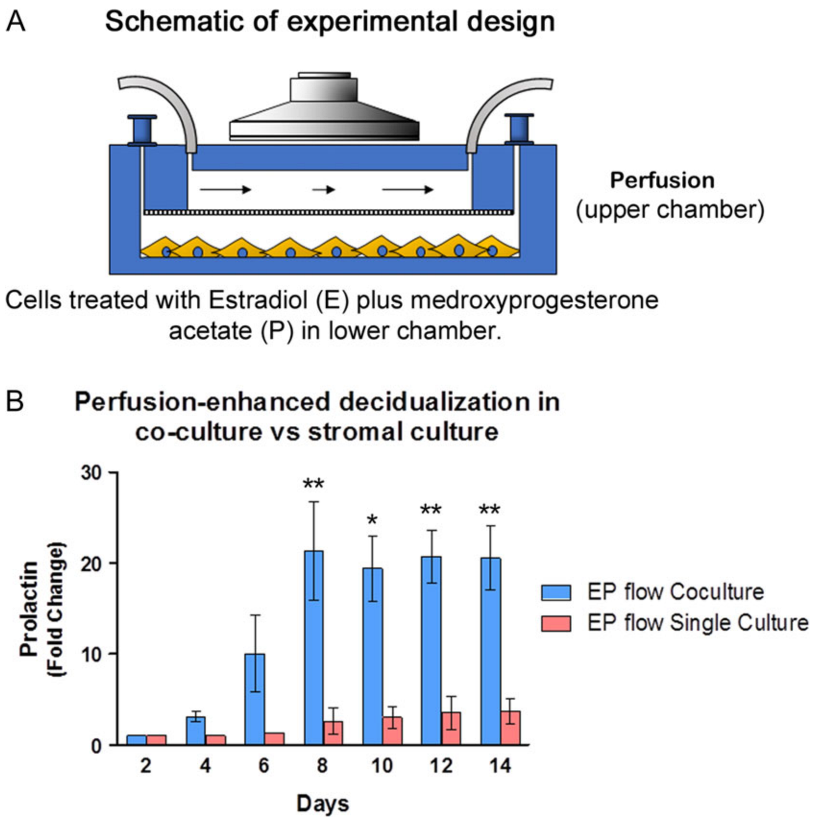

Figure S1 **Perfusion enhanced decidualization is mediated by the endothelial vascular cells**. Endometrial stromal cell decidualization, as determined by prolactin secretion, was observed to be significantly enhanced in the co-culture model with UtMVEC compared to stromal cell single culture perfused with an empty endothelial chamber. ($N = 3$) P values, *<0.05; ** <0.01; ***<0.001.
