# Supplementary material for: Hemodynamic forces enhance decidualization via endothelial-derived prostaglandin E2 and prostacyclin in a microfluidic model of the human endometrium
Source: Hum Reprod. 2019 Feb 21;34(4):702–14. doi: 10.1093/humrep/dez003 (PMC6443116; doi:10.1093/humrep/dez003)
Supplement: Supplementary Figure 2 [file dez003_supplementary_figure_2.pdf]

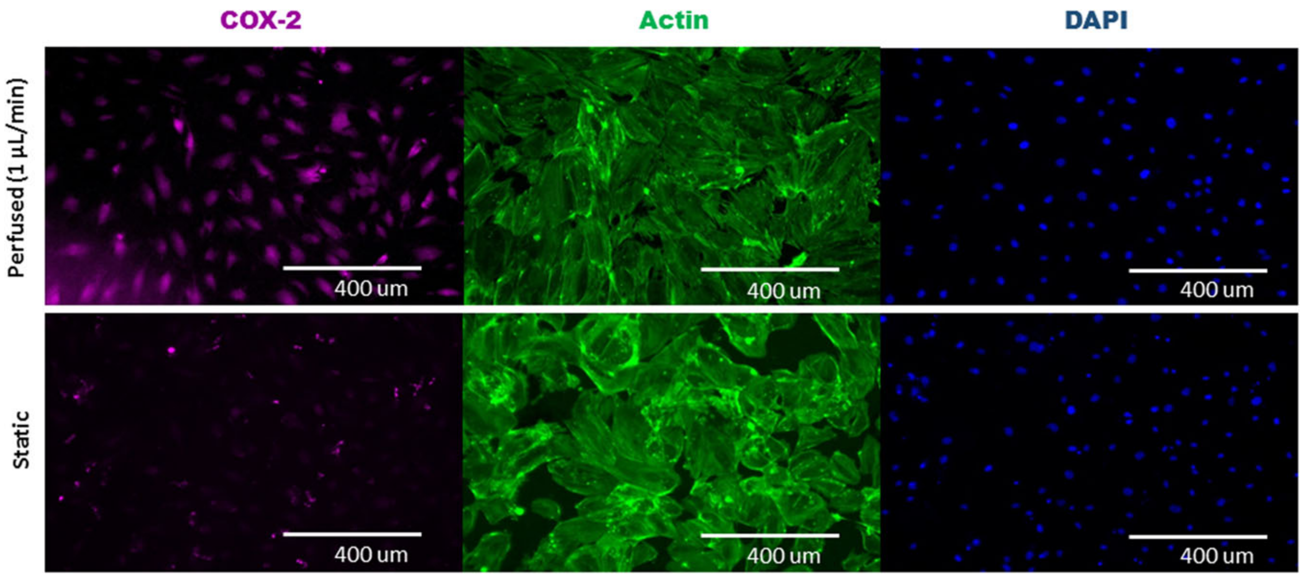

Figure S2 **Immunofluorescence of COX-2 *in vitro* perfused endothelial cells**. Cultured endothelial cells under flow perfusion at 1 μL/min have induced COX-2 (purple) expression and reorganization of actin filaments in the direction of the flow (actin, green, original magnification ×10, all scale bars = 400 μm).
