# Supplementary material for: Hemodynamic forces enhance decidualization via endothelial-derived prostaglandin E2 and prostacyclin in a microfluidic model of the human endometrium
Source: Hum Reprod. 2019 Feb 21;34(4):702–14. doi: 10.1093/humrep/dez003 (PMC6443116; doi:10.1093/humrep/dez003)
Supplement: Supplementary Figure 3 [file dez003_supplementary_figure_3.pdf]

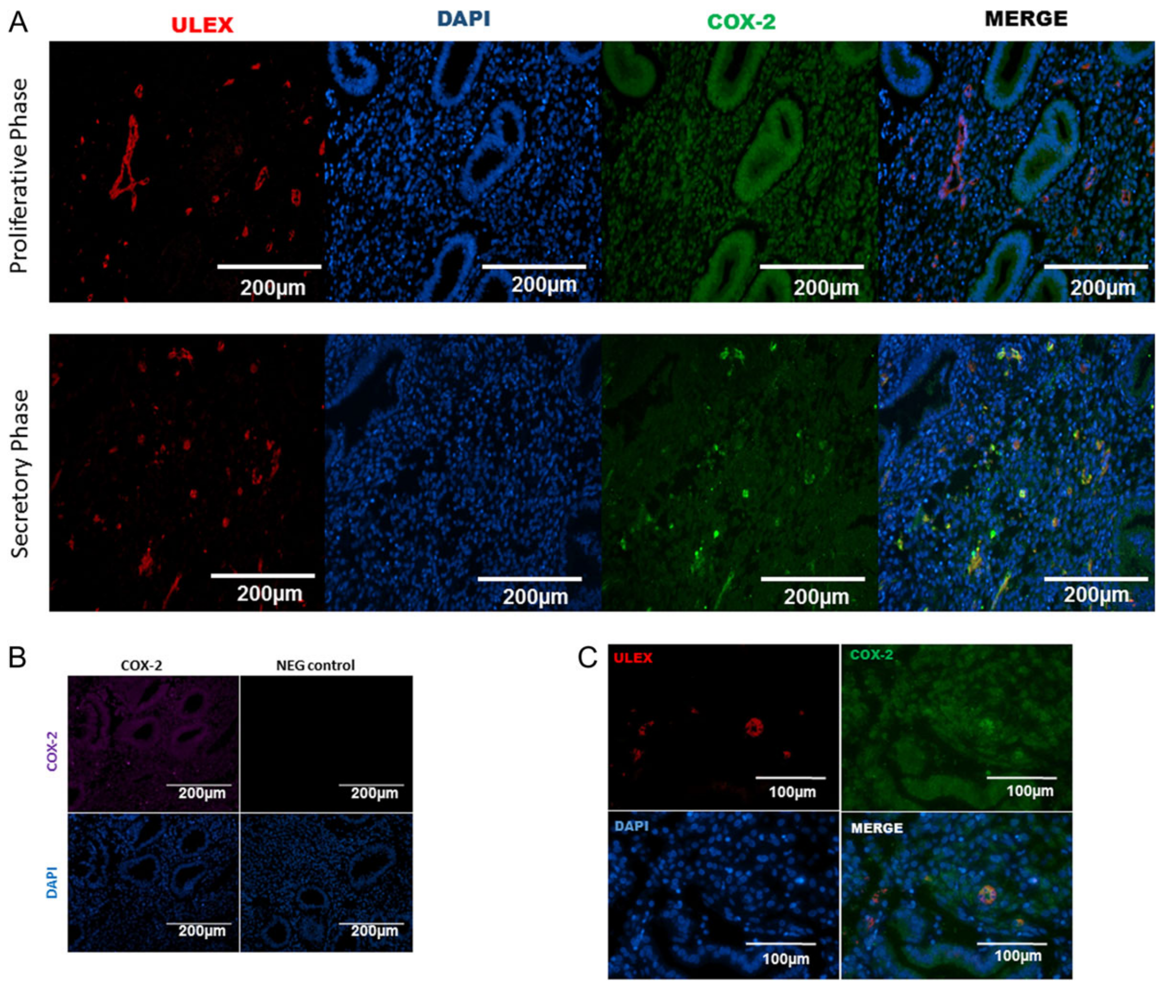

Figure S3 Immunofluorescence of COX-2 in the *ex vivo* human endometrium. (**A**) Co-localization of endothelial cells (ULEX, red) and COX-2 (green) expression in the proliferative and secretory phase of the human endometrium. In the proliferative phase, COX-2 does not localize to the vascular endothelium, but is primarily present in the epithelium and local immune cells. In the secretory phase, co-localization is observed in the terminal spiral arterioles (yellow) (×20, scale = 200 µm). (**B**) Characterization of internal controls in immunofluorescence in tissues (COX-2, purple), (×20, scale = 200 µm). (**C**) Co-localization in secretory phase endometrium, (×40, scale = 100 µm).
