# Supplementary material for: Hemodynamic forces enhance decidualization via endothelial-derived prostaglandin E2 and prostacyclin in a microfluidic model of the human endometrium
Source: Hum Reprod. 2019 Feb 21;34(4):702–14. doi: 10.1093/humrep/dez003 (PMC6443116; doi:10.1093/humrep/dez003)
Supplement: Supplementary Table 1 [file dez003_supplementary_table_si.pdf]

Supplementary Table SI **List of reagents used.**

Item	Details	Cat. number	Source	Working concentration(s)
Supplies				
Blunt-end needle		B25-100	SAI infusion Tech	
Tygon Tubing	0.02'ID × 0.06'OD	EW-06418-02	Tygon	
Poly-L-lysine		p4707	Sigma	
Collagen Type IV		sc29010	Santa Cruz	(10 μg/cm^2)
Antibodies				
PGE_2 receptor 2 (EP2)	Rabbit	101750	Cayman Chemicals	1:100
PGE_4 receptor 4 (EP4)	Rabbit	101775	Cayman Chemicals	1:100
PGD_2 receptor 1 (DP1)	Rabbit	101640	Cayman Chemicals	1:100
PGI_2 receptor (IP)	Mouse	sc515139	Santa Cruz	1:100
Ulex europaeus agglutinin I (ULEX)- Rhodamine	N/A	RL-1062	VECTOR	1:100
hCyclooxygenase-2- AF647	Mouse	sc-376861	Santa Cruz	1:50
ActinGreen™ 488 ReadyProbes™		R37110	ThermoFisher	1 drop/mL
CD31		M0823	DAKO	1:50
Vimentin		ab45939	Abcam	1:400
Goat anti rabbit-AF647	Goat	AnaSpec, Inc	AS-61056-05-H647	1:200
Ready probes GAM-AF594	Goat	R37115	ThermoFisher	1 drop/mL
Biotinylated IgG	Goat	HK335	Biogenex	1 drop/mL
Prostanoid agonists				
Iloprost (Ilo)		18215	Cayman Chemicals	25 μM, 2.5 μM, 1 μM, 500 nM, 250 nM, 100 nM,
Prostaglandin E2 (PGE2)		p0409	Sigma	1 μM, 500 nM 100 nM, 10 nM
Prostaglandin D2 (PGD2)		p5172	Sigma	10 μM, 1 μM
8-Br-cyclic AMP		B7880	Sigma	0.5 mM
Prostanoid antagonists				
Indomethacin	COX-2 inhibtor	I7378	Sigma	100 μM
BAY u3405	DP1 inhibitor	10156	Cayman Chemicals	n/a
CAY10441	IP inhibitor	10005186	Cayman Chemicals	10 μM
AH6809	EP2 inhibitor	A1221	Sigma	20 μM
AH23848	EP4 inhibitor	A8227	Sigma	20 μM
ELISAs				
Prolactin		DY682	R&D	384 well format
Insulin like growth factor binding protein 1 (IGFBP-1)		DY871-05	R&D	384 well format
PGE_2		500141	Cayman Chemicals	96 well format
PGI_2 (Prostacyclin)		ADI-900-025	Enzo	96 well format
